# Non-diagnostic 12-Lead Electrocardiogram (ECG) in an Acute Proximal-to-Mid Right Coronary Artery Occlusion: A Case of Isolated Right Ventricular Infarction

**DOI:** 10.7759/cureus.96144

**Published:** 2025-11-05

**Authors:** Guna Sai Vallapuri, Pankaj V Jariwala, Dilip Gude, Bharat Kumar Reddy K, Sukesh Jangam

**Affiliations:** 1 Cardiology, Yashoda Super Speciality Hospital, Somajiguda, Hyderabad, IND; 2 General Medicine, Yashoda Super Speciality Hospital, Somajiguda, Hyderabad, IND

**Keywords:** acute myocardial infarction, acute right coronary artery occlusion, coronary angiography, echocardiography, non-diagnostic ecg, primary pci, right coronary artery, right-sided leads, right ventricular infarction, st-segment elevation myocardial infarction (stemi)

## Abstract

Proximal-to-mid right coronary artery (RCA) occlusion typically produces inferior ST-segment elevation, yet isolated right ventricular (RV) infarction may appear non-diagnostic on a standard 12-lead electrocardiogram (ECG). A 50-year-old man presented with two hours of chest pain and rising high-sensitivity troponin I levels. The admission 12-lead ECG showed sinus rhythm with left axis deviation and subtle anterior T-wave inversion without inferior ST-segment elevation. Transthoracic echocardiography revealed mild RV dilation and hypokinesis with preserved left ventricular (LV) function. Coronary angiography demonstrated a thrombotic proximal-to-mid RCA occlusion, and primary percutaneous coronary intervention (PCI) restored the Thrombolysis in Myocardial Infarction (TIMI) 3 flow with prompt symptom relief. This case highlights the diagnostic challenge of "ECG-silent" proximal RCA occlusion and emphasizes the role of right-sided leads and early invasive evaluation to avoid delayed reperfusion.

## Introduction

Right ventricular myocardial infarction (RVMI) occurs most commonly with proximal-to-mid right coronary artery (RCA) occlusion and is often underdiagnosed on standard 12-lead electrocardiogram (ECG) because the right ventricular (RV) mass is small and its electrical vectors are easily overshadowed by left ventricular (LV) forces [1,2]. Failure to recognize RVMI can result in inappropriate management, such as nitrate use in preload-dependent RV infarction, and a worsened prognosis [3]. Landmark studies have established that right-sided leads, especially V3R and V4R, significantly improve the diagnostic accuracy [2-4]. We present a patient with angiographically proven proximal-to-mid RCA occlusion who had a non-diagnostic ECG and isolated RV involvement and compare the findings with international benchmarks.

Isolated RVMI accounts for <5% of acute myocardial infarctions (AMIs) and is frequently under-recognized on the standard 12-lead [5-7]. Contemporary guidance recommends obtaining V3R-V6R, particularly V4R, whenever RVMI is suspected (e.g., inferior ST-segment elevation myocardial infarction (STEMI) pattern, hypotension with clear lungs, or atypical 12-lead findings) [6,7].

## Case presentation

A 50-year-old man with hypertension and a 30-pack-year smoking history presented with two hours of retrosternal chest pain radiating to the left arm, diaphoresis, and nausea. The patient's vital signs on presentation were stable, with a blood pressure of 110/70 mmHg, a heart rate of 58 beats per minute, a respiratory rate of 18 breaths per minute, and an oxygen saturation of 97% on room air. On examination, the lungs were clear, with mild jugular venous distension and no pulmonary edema on auscultation and imaging review. Laboratory evaluation showed an elevated high-sensitivity troponin I that peaked at 1.8 ng/mL (normal <0.04 ng/mL). Other routine laboratory values were within reference ranges and non-contributory.

Past medical history included hypertension treated with an angiotensin-converting enzyme (ACE) inhibitor; there was no prior antiplatelet or statin therapy.

Timeline and management sequence

Serial ECGs were obtained at zero hours (admission), ~2 hours (pre-percutaneous coronary intervention (PCI)), ~3 hours (post-PCI), ~24 hours (early ward), and ~96 hours (predischarge). High-sensitivity troponin I was sampled at zero hours, three hours, and 12 hours (peak 1.8 ng/mL). The decision to proceed with invasive angiography was made ~90 minutes after presentation based on persistent symptoms with rising biomarkers despite a non-diagnostic 12-lead ECG.

**Figure 1:**
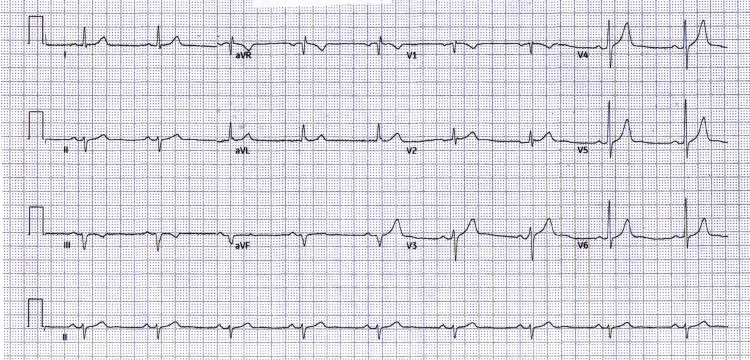
Admission (non-diagnostic ECG) Admission ECG: sinus bradycardia, left axis deviation, no inferior ST-segment elevation, and subtle anterior T-wave inversion in V1-V2. ECG: electrocardiogram; aVR: augmented vector right; aVL: augmented vector left; aVF: augmented vector foot

**Figure 2:**
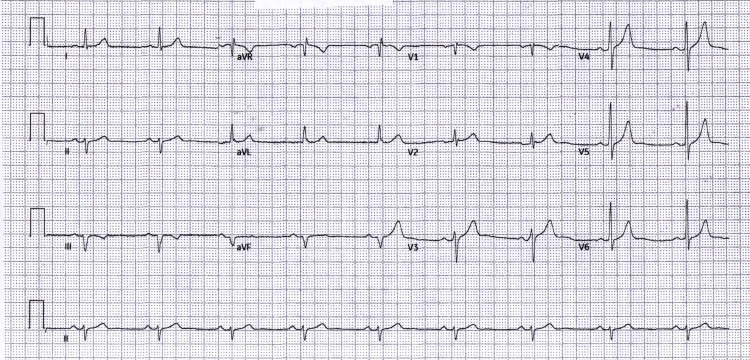
Pre-PCI repeat (unchanged) Pre-PCI repeat ECG: unchanged and persistently non-diagnostic despite ischemic symptoms/biomarkers. PCI: percutaneous coronary intervention; ECG: electrocardiogram; aVR: augmented vector right; aVL: augmented vector left; aVF: augmented vector foot

The 12-lead ECG at admission (Figure 1) showed sinus bradycardia with left axis deviation and no inferior ST-segment elevation; only subtle anterior T-wave changes (flattening/inversion in V1-V2) were observed. The pre-procedural repeat (Figure 2) was unchanged, confirming a persistently non-diagnostic 12-lead despite ongoing ischemic symptoms and rising high-sensitivity troponin levels. Pre-procedure management included aspirin, a P2Y12 inhibitor, anticoagulation with heparin, and analgesia.

**Figure 3:**
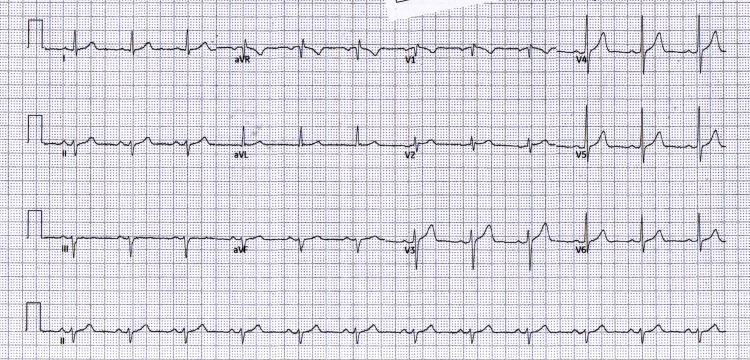
Immediate post-PCI ECG Immediate post-PCI ECG: clinical improvement after RCA stenting and no new ischemic changes. PCI: percutaneous coronary intervention; RCA: right coronary artery; ECG: electrocardiogram; aVR: augmented vector right; aVL: augmented vector left; aVF: augmented vector foot

**Figure 4:**
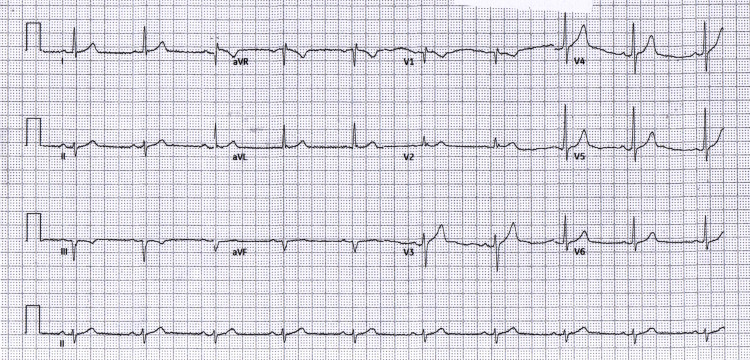
Early follow-up (ward) ECG Early follow-up (ward) ECG: sinus rhythm ~60-65 bpm; no inferior STE; anterior T-waves less conspicuous. ECG: electrocardiogram; STE: ST-segment elevation; aVR: augmented vector right; aVL: augmented vector left; aVF: augmented vector foot

**Figure 5:**
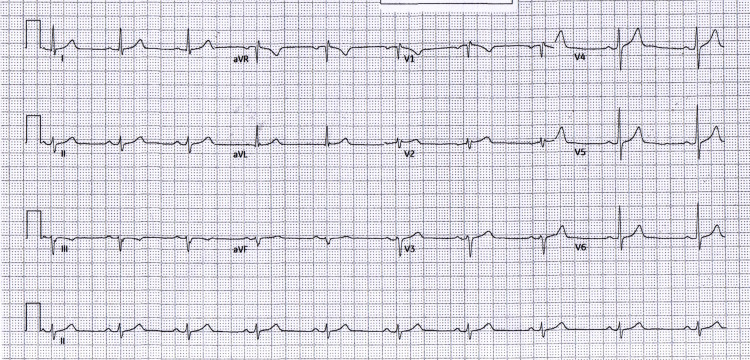
Predischarge ECG Predischarge ECG: stable tracing without inferior Q-waves; automated comments (e.g., incomplete RBBB/poor R-wave progression) are non-diagnostic in this setting. ECG: electrocardiogram; RBBB: right bundle branch block; aVR: augmented vector right; aVL: augmented vector left; aVF: augmented vector foot

Following primary PCI of the proximal-to-mid RCA, the immediate post-PCI ECG (Figure 3) showed no new ischemic changes, and the patient's pain resolved. Early ward and predischarge tracings (Figure 4 and Figure 5) remained free of inferior ST-segment elevation and showed no evolution to inferior Q-waves, consistent with an isolated RV infarction rather than classic inferior STEMI.

Right-sided leads recorded immediately pre-PCI demonstrated ~1 mm ST-segment elevation in V3R/V4R (supporting RV involvement; not shown in Figure 1), aligning with the echocardiographic findings. This corroborated the non-diagnostic 12-lead pattern and supported RV involvement in the context of proximal-to-mid RCA occlusion. The right-sided tracing is archived in the clinical record and summarized here to preserve de-identification.

Transthoracic echocardiography demonstrated a preserved LV ejection fraction (~58%) without LV wall motion abnormality, but mild RV dilation and hypokinesis. Representative echocardiographic frames were unavailable for publication, and the findings are therefore summarized narratively.

**Figure 6:**
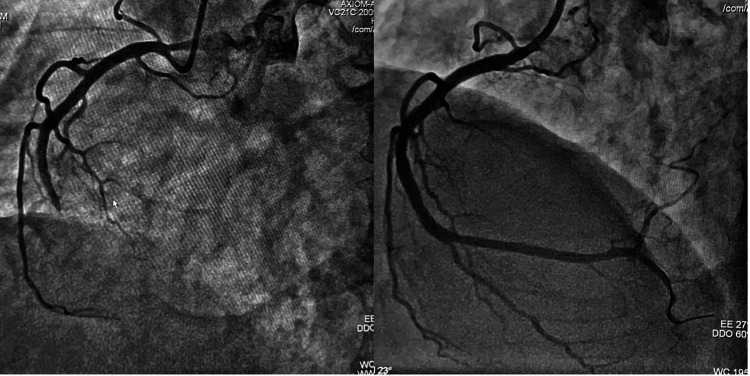
Coronary angiography Coronary angiography demonstrating the complete thrombotic occlusion of the proximal-to-mid RCA (TIMI 0 flow) and successful stenting with the restoration of TIMI 3 flow. These findings confirmed isolated right ventricular involvement despite preserved left ventricular function. Echocardiographic frames were unavailable; findings are described narratively in the text. The left coronary system was angiographically normal (image not included for brevity). RCA: right coronary artery; TIMI: Thrombolysis in Myocardial Infarction

Urgent angiography revealed a thrombotic occlusion in the proximal-to-mid RCA (Thrombolysis in Myocardial Infarction (TIMI) 0 flow) with an angiographically normal left system (image not included). PCI was then performed. Primary PCI with a drug-eluting stent restored TIMI 3 flow and relieved pain immediately (Figure 6).

The patient remained stable without arrhythmias or shock and was discharged on day 5 with dual antiplatelet therapy, statins, ACE inhibitors, and beta-blockers. He was asymptomatic at the one-month follow-up visit.

To document the non-diagnostic pattern over time, serial tracings at admission, pre-PCI, immediate post-PCI, early follow-up, and predischarge are summarized in Table 1 and shown in Figures 1-5. The clinical course is presented alongside ECG evolution and echo notes to maintain continuity with management decisions.

**Table 1:** Serial clinical status, ECG evolution, echocardiography, and management The table shows that inferior STE never appeared despite angiographic occlusion, confirming a non-diagnostic pattern linked to isolated RV involvement. ECG: electrocardiogram; STE: ST-segment elevation; PCI: percutaneous coronary intervention; TIMI: Thrombolysis in Myocardial Infarction; LV: left ventricle/ventricular; RV: right ventricle/ventricular; EF: ejection fraction; DAPT: dual antiplatelet therapy

Time point	Key clinical findings	ECG evolution	Hemodynamics/echo	Management
Admission	Chest pain, bradycardia; no inferior STE	Non-diagnostic; subtle changes	RV dilation; preserved LV	Supportive care and catheterization planning
Pre-PCI (day 1)	Worsening chest discomfort	Subtle V3R/V4R STE	RV dysfunction confirmed	PCI initiated
Immediate post-PCI	Chest pain relief	Resolution of STE	TIMI 3 flow; RV recovery	DAPT + heparin
Day 3 (early ward)	Clinically stable	T-wave inversions evolving	LV preserved	Mobilization, risk stratification
Day 5 (predischarge)	Asymptomatic	ST segments normalized	RV improving	Discharged on DAPT, statins, beta-blockers
1-month follow-up	No recurrence of angina; clinically active	ECG near-normal	Echo: RV near-normal, EF preserved	Continued medical therapy

## Discussion

Mechanisms of a "false-negative" ECG

Classical literature describes several mechanisms for the absence of inferior ST-segment elevation in proximal-to-mid RCA occlusions [1-5]. The results are presented in Table 2.

**Table 2:** Mechanisms for non-diagnostic ECG in proximal-to-mid RCA occlusion These mechanisms explain why our patient's ECG appeared "benign" despite proximal-to-mid RCA occlusion and justify right-sided leads in suspected RV infarction. Data summarized from [1-5]. RCA: right coronary artery; ECG: electrocardiogram; PDA: posterior descending artery; LV: left ventricle/ventricular; RV: right ventricle/ventricular; STEMI: ST-segment elevation myocardial infarction

Mechanism	Explanation	Clinical relevance
Isolated RV infarction	Small RV mass generates weak injury currents	Can produce subtle anterior T-wave changes only [1,3]
Electrical masking	LV forces dominate	False-negative inferior leads; need right-sided leads [2]
Collateral circulation	Chronic collaterals blunt ischemic currents	Attenuates ST-segment elevation [5]
PDA sparing	Inferior wall preserved	Mimics unstable angina rather than STEMI [4]

Comparison with inferior STEMI

As inferior STEMI and isolated RVMI overlap clinically, we constructed a side-by-side comparison (Table 3) to highlight the distinguishing features. Table 3 contrasts classic inferior STEMI with isolated RV infarction to emphasize diagnostic and management differences.

**Table 3:** Inferior STEMI vs isolated RV infarction This table highlights the distinct clinical profile of isolated RVMI, emphasizing that therapy must be tailored to preload sensitivity and RV-specific complications. Data compiled from [1-5]. ECG: electrocardiogram; STEMI: ST-segment elevation myocardial infarction; aVL: augmented vector left; aVF: augmented vector foot; LVEF: left ventricular ejection fraction; AV: atrioventricular; LV: left ventricle/ventricular; RV: right ventricle/ventricular; PDA: posterior descending artery; RCA: right coronary artery; RVMI: right ventricular myocardial infarction

Domain	Inferior STEMI	Isolated RV infarction (this case)
ECG	ST-segment elevation II, III, aVF; reciprocal in I, aVL	Non-diagnostic 12-lead; V4R more sensitive [2-4]
Echo	Inferior wall hypokinesis; ↓LVEF	RV dilation/hypokinesis; preserved LV
Hemodynamics	Pulmonary congestion possible	Preload-dependent; nitrates contraindicated [3]
Complications	AV block, LV dysfunction	RV failure, bradyarrhythmias
Angiography	RCA/PDA culprit	RCA proximal to RV branches

Literature-benchmarked diagnostic analytics and outcomes

**Figure 7:**
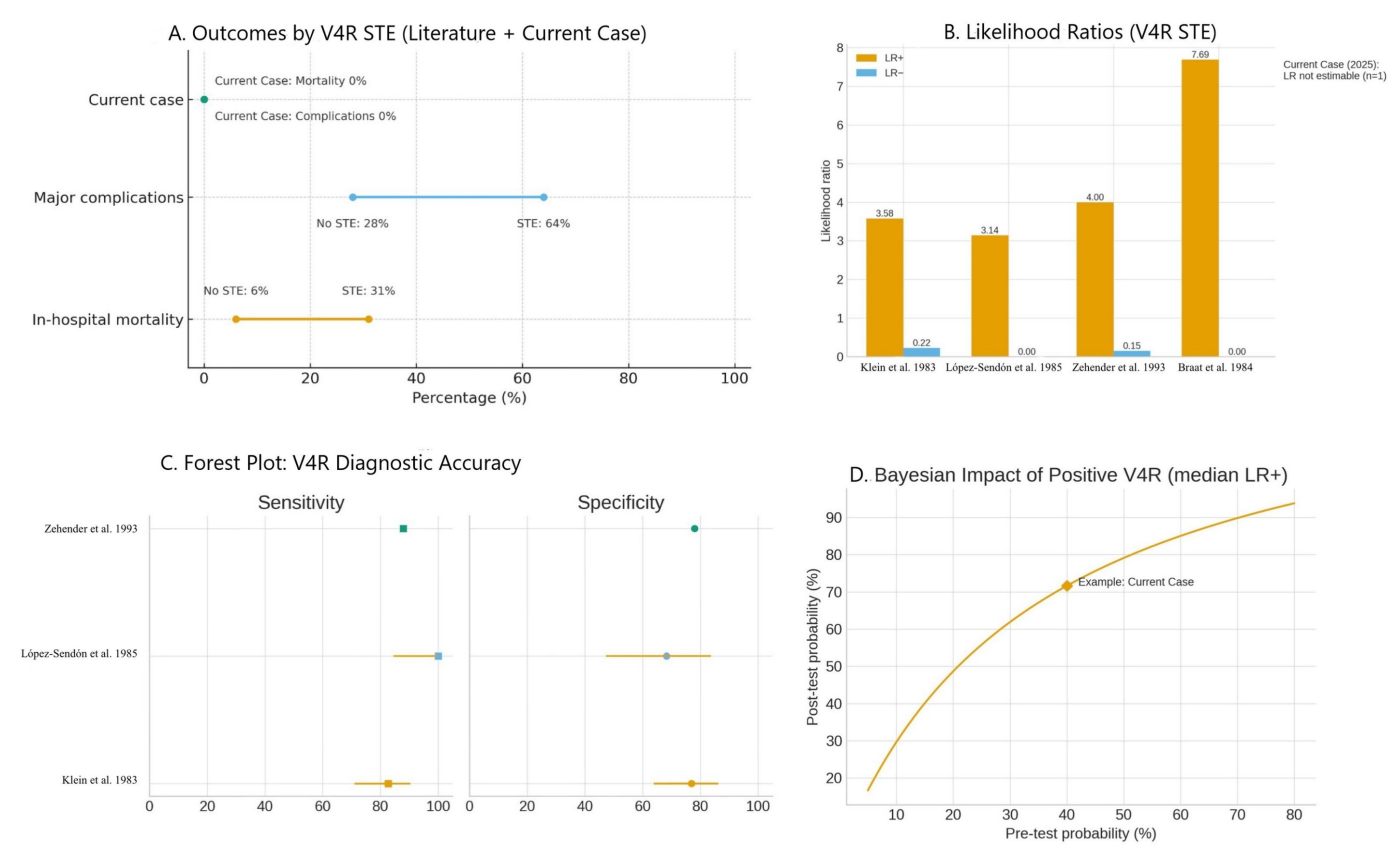
Four-panel evidence synthesis Panel A (outcomes dumbbell): mortality and complication rates from Zehender et al. [4] (no STE: 6%/28%; STE: 31%/64%) contrasted with our case (0% for both), emphasizing the outcome risk when V4R STE is missed. Panel B (LRs): LR+ and LR– derived from benchmark studies (Table 4), showing how diagnostic yield can shift from pre- to post-test probability in suspected RV infarctions. Panel C (forest plot): sensitivity and specificity estimates with Wilson 95% CIs from landmark studies summarizing diagnostic accuracy across the literature (Table 4). Panel D (Bayesian curve): post-test probability transformation using the median LR+, illustrating how Bayesian updating increases the diagnostic certainty when V4R is positive. This figure integrates the diagnostic accuracy and outcome data from the literature, overlaying the current case to emphasize the gap between classical benchmarks and real-world atypical presentations. Panel B-D values come from Table 4 (LRs, CI, median LR+). Benchmark data for panels A-D derived from [1-4]. STE: ST-segment elevation; RV: right ventricle/ventricular; LR: likelihood ratios

To strengthen the context of this case, we compared our findings against international benchmarks for right-sided leads. Figure 7 synthesizes diagnostic performance and outcomes using multidimensional and evidence-based visualizations. These figures allow direct comparison between our case and established literature, highlighting both the diagnostic limitations of the standard 12-lead and the prognostic implications of missed RV infarction. The tables and figures are qualitative benchmarks intended to contextualize a single case rather than statistical comparisons.

International benchmarks for V4R

To benchmark our case internationally, we summarized the diagnostic accuracy metrics for the V4R from the landmark series (Table 4). Metrics were abstracted from primary sources [1-4] to align our case with established ranges.

**Table 4:** International benchmarks for V4R in suspected RV infarction Benchmarks were extracted directly from published series [1-4] and are presented for qualitative context only (not statistical inference). The LRs in Figure 7B are derived as LR+ = Sens/(1 - Spec) and LR- = (1 - Sens)/Spec. The Bayesian curve (Figure 7D) was applied to the median LR+. The radar plot (Figure 7) normalizes each axis to 0-1, based on these ranges. LR: likelihood ratios; NPV: negative predictive value; RCA: right coronary artery; STE: ST-segment elevation; RV: right ventricle/ventricular

Study	Sample (n)	Sensitivity (%)	Specificity (%)	Notes
Klein et al., 1983 [1]	110	82.7	76.9	Early V4R recognition; high NPV
López-Sendón et al., 1985 [2]	43	100	68.2	V3R/V4R outperforms inferior leads
Braat et al., 1984 [3]	62	~100	87	V4R ≥1 mm predicts proximal RCA
Zehender et al., 1993 [4]	200	88	78	V4R STE predicts RV involvement and higher mortality
Current case, 2025	1	-	-	Proximal RCA occlusion with non-diagnostic 12-lead

Diagnostic insights and clinical relevance

The absence of inferior ST-segment elevation across five serial tracings (Figures 1-5) despite angiographically proven proximal-to-mid RCA occlusion underscores the electrical masking of RV injury and the risk of false-negative 12-lead interpretations. Taken together with the angiographic result, this sustained pattern is most consistent with isolated RV involvement rather than classic inferior STEMI.

This case demonstrates how proximal-to-mid RCA occlusion may present without inferior ST-segment elevation when ischemia is RV dominant. These mechanisms include vector masking, small RV mass, collaterals, and PDA sparing (Table 2) [1-5].

Landmark studies have confirmed the diagnostic utility of right-sided leads. Klein et al. [1] first established their value; López-Sendón et al. [2] and Braat et al. [3] showed nearly universal sensitivity; and Zehender et al. [4] linked V4R positivity with mortality. Our patient reinforced these findings by presenting a complementary scenario, RCA occlusion with a non-diagnostic 12-lead, underscoring why V4R is pivotal.

Our figures further contextualize the case: the radar plot (Figure 7) highlights diagnostic weakness of the 12-lead, the Bayesian curve (Figure 7D) translates LR+ to clinical probability, and the dumbbell plot (Figure 7A) illustrates the prognostic importance of V4R detection. Collectively, these visuals link published benchmarks to bedside decisions in suspected RVMI and are intended as teaching aids rather than statistical analyses.

Global Incidence Context

Isolated RV infarction accounts for <5% of all AMIs, but its prevalence varies by population and is likely under-reported due to the underuse of right-sided leads [6,7]. This gap reinforces the need to record V3R/V4R when presentations or 12-lead findings are atypical [1-4,6,7].

Incremental Knowledge

Unlike previous studies where V4R STE was often present [1-3], our case showed complete proximal-to-mid RCA occlusion without diagnostic changes, underscoring the limitations of relying solely on the 12-lead. This highlights the limitation of relying only on the standard 12-lead when RV involvement predominates [1-4,6,7].

Limitations

As this was a single case, the findings cannot be generalized. Advanced imaging such as cardiac magnetic resonance imaging (MRI) was not performed; however, echocardiography and angiography provided sufficient confirmation.

Clinical Message

A non-diagnostic ECG must not defer angiography when clinical suspicion and biomarkers suggest an acute occlusion. The management of isolated RVMI should avoid the use of preload-reducing drugs and prioritize early revascularization [3,6,7].

## Conclusions

Proximal-to-mid RCA occlusion can masquerade as a "normal" ECG when RV involvement predominates. Right-sided leads in the appropriate scenarios (inferior STEMI pattern, hypotension with clear lungs, or atypical 12-lead findings), together with early invasive evaluation and RV-focused management, are essential for avoiding missed diagnoses and adverse outcomes. In patients with persistent symptoms and rising biomarkers despite a non-diagnostic 12-lead, obtaining V3R/V4R and pursuing timely angiography are key steps aligned with guidance.
